# The Generation of Live Offspring from Vitrified Oocytes

**DOI:** 10.1371/journal.pone.0021597

**Published:** 2011-06-27

**Authors:** L. Gabriel Sanchez-Partida, Richard D. W. Kelly, Huseyin Sumer, Camden Y. Lo, Rotem Aharon, Michael K. Holland, Moira K. O'Bryan, Justin C. St. John

**Affiliations:** 1 Centre for Reproduction and Development, Monash Institute of Medical Research, Monash University, Victoria, Australia; 2 Australian Phenomics Network, Monash Animal Research Platform, Monash University, Victoria, Australia; 3 Mitochondrial and Reproductive Genetics Group, Clinical Sciences Research Institute, Warwick Medical School, University of Warwick, Coventry, United Kingdom; 4 School of Mathematical Sciences, Monash University, Victoria, Australia; 5 Monash Micro Imaging – MHTP, Monash Institute of Medical Research, Clayton, Victoria, Australia; 6 School of Veterinary Sciences, The University of Queensland, St. Lucia Campus, Brisbane, Queensland, Australia; 7 The Department of Anatomy and Developmental Biology, Monash University, Victoria, Australia; Wellcome Trust Centre for Stem Cell Research, United Kingdom

## Abstract

Oocyte cryopreservation is extremely beneficial for assisted reproductive technologies, the treatment of infertility and biotechnology and offers a viable alternative to embryo freezing and ovarian grafting approaches for the generation of embryonic stem cells and live offspring. It also offers the potential to store oocytes to rescue endangered species by somatic cell nuclear transfer and for the generation of embryonic stem cells to study development in these species. We vitrified mouse oocytes using a range of concentrations of trehalose (0 to 0.3 M) and demonstrated that 0.1 and 0.3 M trehalose had similar developmental rates, which were significantly different to the 0.2 M cohort (P<0.05). As mitochondria are important for fertilisation outcome, we observed that the clustering and distribution of mitochondria of the 0.2 M cohort were more affected by vitifrication than the other groups. Nevertheless, all 3 cohorts were able to develop to blastocyst, following in vitro fertilisation, although developmental rates were better for the 0.1 and 0.3 M cohorts than the 0.2 M cohort (P<0.05). Whilst blastocysts gave rise to embryonic stem-like cells, it was apparent from immunocytochemistry and RT-PCR that these cells did not demonstrate true pluripotency and exhibited abnormal karyotypes. However, they gave rise to teratomas following injection into SCID mice and differentiated into cells of each of the germinal layers following in vitro differentiation. The transfer of 2-cell embryos from the 0.1 and 0.3 M cohorts resulted in the birth of live offspring that had normal karyotypes (9/10). When 2-cell embryos from vitrified oocytes underwent vitrification, and were thawed and transferred, live offspring were obtained that exhibited normal karyotypes, with the exception of one offspring who was larger and died at 7 months. We conclude that these studies highlight the importance of the endometrial environment for the maintenance of genetic stability and thus the propagation of specific genetic traits.

## Introduction

Oocyte cryopreservation offers a number of potential benefits for assisted reproductive technologies, the treatment of infertility and biotechnology. For example, it reduces the necessity for women undergoing chemotherapy to have to rely on the highly invasive approach of ovarian freezing and subsequent grafting to rescue oocytes [1]. It also reduces the need for women to undergo repeated hyper-stimulation protocols as oocytes, rather than embryos, could be stored [2]. Furthermore, oocyte cryopreservation is particularly attractive for women in countries where embryo freezing is restricted through legislation and emphasis lies on gamete freezing [3].

Cryopreservation also offers the opportunity to store oocytes from endangered species that can then be either fertilised with fresh or cryopreserved sperm from the same species or sub-species or used as recipients for somatic cell nuclear transfer (SCNT) to propagate the desired genetic material [4]. The resultant embryos could then be transferred to the mother or a surrogate to produce live offspring or cultured in vitro to derive embryonic stem (ES) cells. Characteristically, ES cells derived from the same embryo are genetically identical and self-renew [5]. They are also pluripotent in nature, which confers the potential to differentiate into all cell types of the body and provides an excellent resource for studying cellular differentiation and development, especially when the gametes used to generate these cells are from near extinct or endangered species.

Nevertheless, the reestablishment of a species is often dependent on the use of a somatic cell transferred into the enucleated oocyte from a closely related species or subspecies [6]. However, a number of genetic and epigenetic abnormalities arise from SCNT due to the somatic nature and epigenetic memory associated with these cells and, as a result, the process is highly reliant on the reprogramming of the somatic cell by the recipient oocyte's cytoplasm [7]. The use of fertilised cryopreserved oocytes would allay the use of this more genetically divergent approach and promote chromosomal and mitochondrial genetic identity and integrity. In this instance, sperm stored from the same species would be used to fertilise cryopreserved oocytes, which would then be cultured to the blastocyst stage from which ES cells could be harvested. This would provide an unlimited source of pluripotent ES cells for subsequent rounds of nuclear transfer and would ensure that the oocyte would not need to reprogramme the transferred cell. In turn, this would increase nuclear transfer outcome and reduce the number of abnormalities arising from reprogramming. Common approaches to oocyte cryopreservation include: i) slow-freeze and rapid thaw; and ii) rapid freezing and warming, i.e. vitrification. Both approaches are highly dependent upon cryoprotectant agents (CPA) that protect oocytes from damage during the freezing process. Currently, these approaches necessitate the injection of cryoprotectants into the oolemma [8], laser ablation of the zona pellucida to promote sperm penetration [9] or intracytoplasmic sperm injection [10], [11]. In terms of cryobiology, temperature changes during freezing damage the structural integrity of the oocyte, which can result in oocyte activation and parthenogenesis [12]. Furthermore, cryopreservation can lead to premature extrusion of corticle granules resulting in zona hardening [13]; microtubular spindle injury [14] and condensed chromosomes [15] resulting in, for example, atypical haplotypes. It is likely that cryopreservation will affect mitochondrial integrity, which will reduce the oocyte's stores of ATP and affect oocyte function. Indeed, mitochondrial function is vital to oocyte competency [16] and ATP, generated through oxidative phosphorylation (OXPHOS) in the mitochondria, rather than cytoplasmic glycolysis, is essential to mediate fertilisation and post-fertilisation events and the first cell division [17]. Furthermore, insufficient numbers of mitochondria in the metaphase II oocyte often result in preimplantation development arrest [18]. Collectively or individually, these outcomes will reduce the oocyte's potential to undergo and complete fertilisation and lead to successful and normal development.

In the mouse, modified slow-freezing protocols, with increased sucrose and the replacement of sodium with choline, have resulted in fetal/live birth rates of up to 47.6% [9]. On the other hand, vitrification of mouse oocytes in a G1.2 medium [19] without NaHCO_3_ but supplemented with HEPES and using a nylon loop system, resulted in fetal rates of 37.9% [20]. Although the cryoprotective action of sucrose is well-documented, other CPAs such as trehalose and lactose are more able to stabilise membranes in an aqueous environment than sucrose [21] and would thus provide post-warmed cryopreserved oocytes with minimum intra- and extra-cellular disruption. This would most likely reduce the need for intracytoplasmic sperm injection (ICSI) or zona ablation following warming and promote the maintenance of the oocyte's structural integrity. Nevertheless, the inclusion of sucrose allows the toxic effects of DMSO, a key CPA, to be buffered [22] thus adding to the overall protection provided to the oocyte.

We have set out to demonstrate that oocyte vitrification using trehalose, in combination with sucrose, is a viable alternative to embryo freezing and ovarian grafting approaches for the generation of ES cells and live offspring. We have demonstrated that vitrified oocytes can be fertilized in vitro where motile sperm can penetrate the zona pellucida and propagate syngamy, instead of relying on invasive processes such as ICSI, injection of cryoprotectants into the oolemma or laser ablation of the zona pellucida. We have demonstrated that embryos obtained from vitrified oocytes were able to generate live offspring. Similarly, when 2-cell embryos from vitrified oocytes were subjected to a further round of vitrification, live offspring were also obtained following warming and embryo transfer. However, the long-term culture of ES cells derived from vitrified oocytes suggests that they are more susceptible to abnormal karyotypes. The latter highlights the importance of the endometrial environment for the maintenance of genetic stability and thus the propagation of specific genetic traits.

## Results

### Vitrification of oocytes

**Figure 1:**
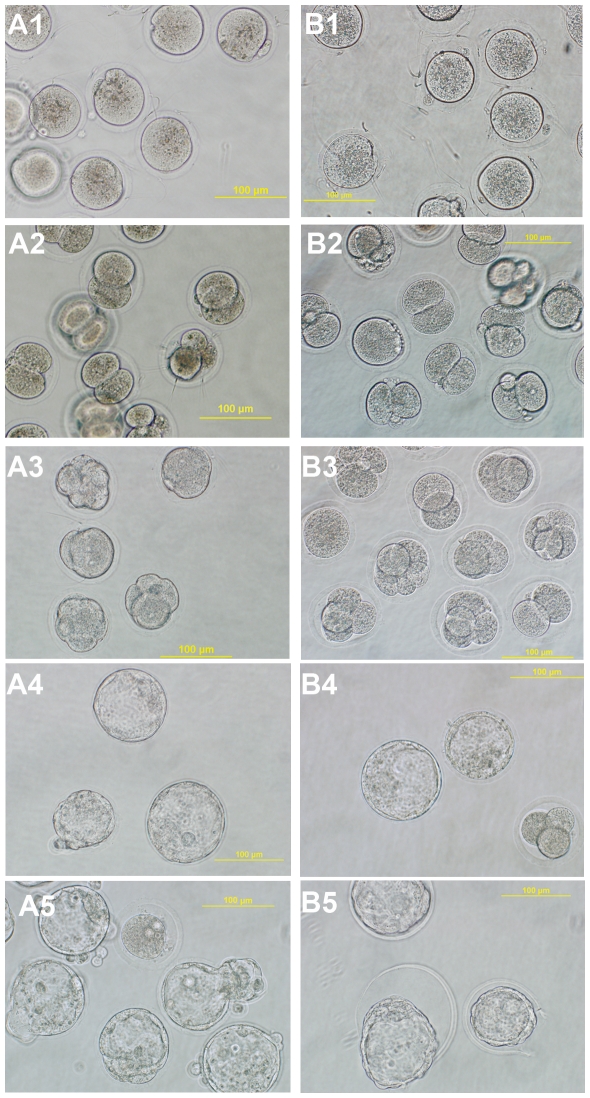
Following in vitro fertilization and culture, both fresh (A1) and vitrified oocytes in 0.1 M trehalose (B1) were capable of developing to the two-cell (A2; B2), four-cell (A3; B3) and blastocyst (A4; B4) stages, respectively. Furthermore, blastocysts were able to hatch (A5; B5) without chemical or mechanical assistance and some were used to derive embryonic stem cells.

In order to derive viable ESCs from vitrified oocytes, we generated a series of blastocysts from oocytes treated with a sucrose-DMSO-EG-based vitrification solution containing a range of trehalose concentrations. In this respect, oocytes were vitrified with one of 0 (control), 0.1, 0.2 and 0.3 M trehalose concentrations and following warming, they were either cultured in fertilisation medium or fertilised with fresh epidydimal sperm and cultured in vitro to blastocyst (see Fig. 1). Parthenogenic activation was not observed when fresh or warmed oocytes were cultured in either mT6 and KSOMaa, as described for the *in vitro* fertilisation (IVF) experiments. Significantly more oocytes treated with 0.1 and 0.3 M trehalose survived post-warming recovery than those treated with 0.2 M trehalose (P<0.05; see Table 1). The 0.1 and 0.3 M trehalose treated oocytes also exhibited recovery rates similar to non-trehalose treated oocytes. Similarly, a higher number of 0, 0.1 or 0.3 M trehalose treated oocytes successfully underwent IVF and progressed to the 2-cell stage than the 0.2 M cohort. Again, significantly more 0.1 and 0.3 M treated cohorts developed to blastocyst (see Fig. 1) than the 0.2 M cohort (P<0.05). In contrast, blastocyst rates were significantly lower for the vitrified oocytes when compared with embryos produced with fresh oocytes (Table 1).

**Table 1:** Survival, two-cell and blastocyst rates for vitrified mouse oocytes following IVF.

Treatment(M of Trehalose)	Vitrified Oocytes	Post-warming survival(%)	Oocytes in culture post IVF (%)	Two-cell(%)	Blastocyst (%)
Fresh	110 (not vitrified)	N/A	105 (95.5)	84 (76.4)	82 (74.5)**
0	82	70 (85.4)	64 (78.0)	61 (74.4)	32 (39.0)
0.1	139	114 (82.0)	89 (64.0)	77 (55.4)	62 (44.6)
0.2	85	32 (37.2)*	25 (29.4)*	17 (20.0)*	5 (5.9)*
0.3	99	85 (85.9)	63 (63.6)	50 (50.5)	39 (39.4)

* Fisher's test significantly lower than other treatments (P<0.05);

** Fisher's test significantly higher than other treatments within the same column (P<0.001). IVF Data collected from 3 replicates.

**Figure 2:**
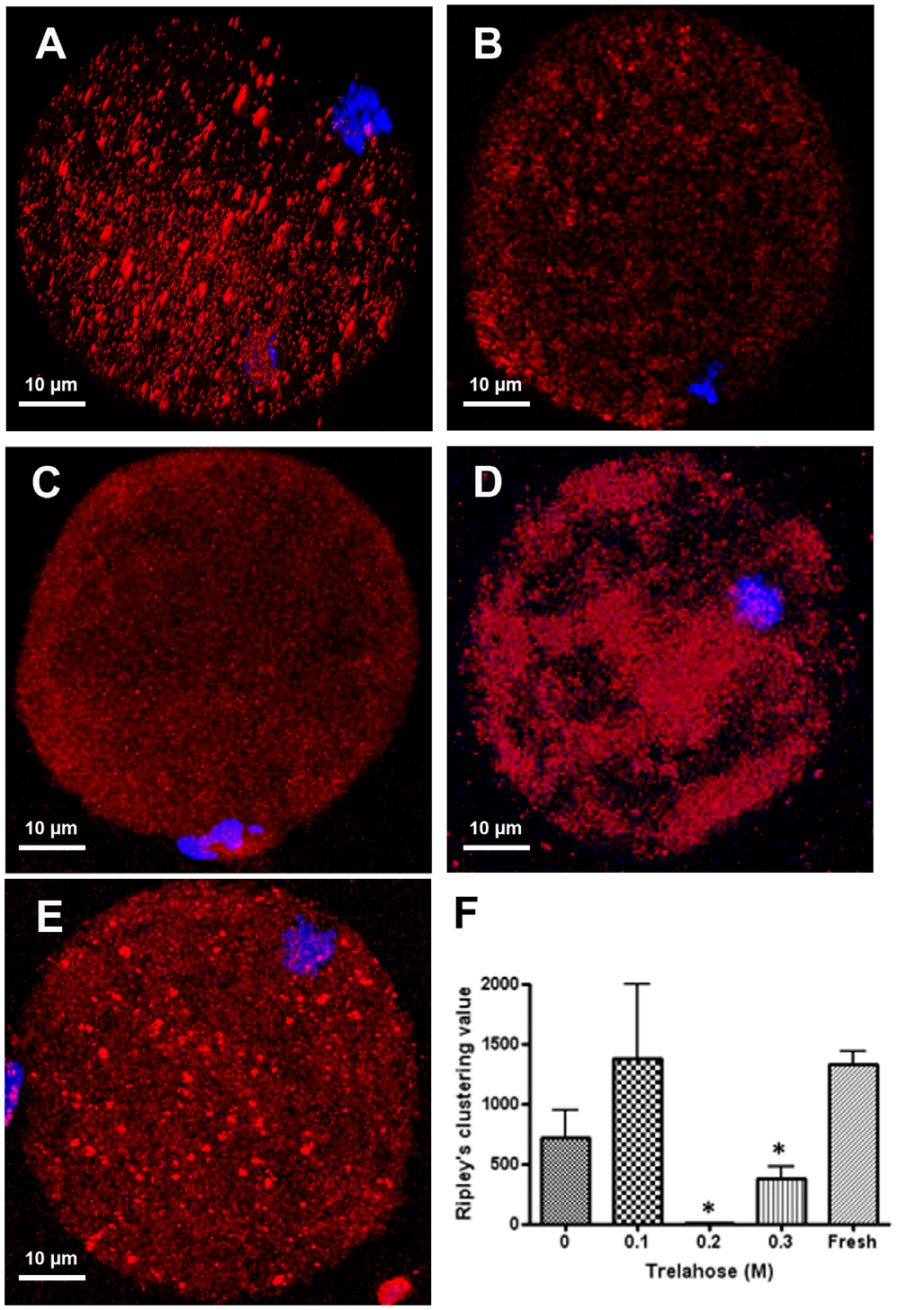
Assessment of mitochondrial integrity in fresh (A) or vitrified mouse oocytes in the presence of 0 (B), 0.1 (C), 0.2 (D) or 0.3 (E) trehalose labelled with Deep MitoTracker Red. Nuclei were stained with DAPI. The level of clustering of mitochondria (F) at the distance criterion (r) of 5 µm, displayed as mean ± SEM. Treatment groups that were significantly different (p<0.05) to that of Fresh oocytes are denoted by *.

As significantly fewer oocytes from the 0.2 M trehalose cohort survived post-warming, we determined whether the vitrification process affected the distribution of mitochondria following warming. We stained oocytes from each cohort with MitoTracker Deep Red FM (Fig. 2A–E) and assessed the distribution of mitochondrial clustering. The clustering was similar for the fresh, 0 and 0.1 M treated groups (Fig. 2A;B;C;F), although for the 0.3 M treatment it was significantly lower (P<0.05; Fig. 2E;F). The values for the 0.2 M cohort demonstrated very little clustering relative to the fresh oocyte population (P<0.05; Fig. 2D;F).

**Figure 3:**
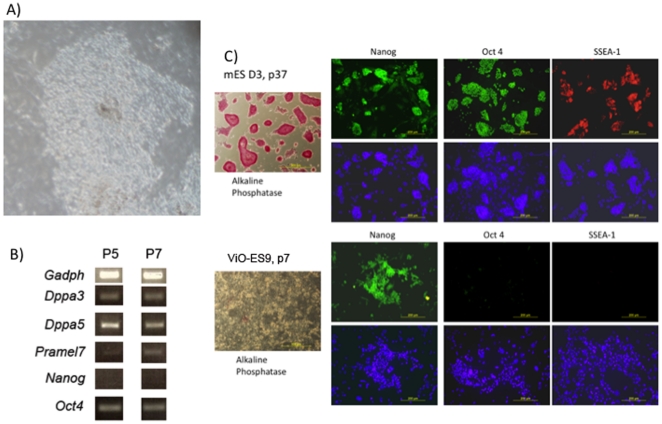
Expression of pluripotent markers in ViO-ES9 cells: ES cell colonies derived from vitrified oocytes (A). RT-PCR amplification of *Oct4*, *Dppa3*, *Dppa5*, *Pramel7* and *Nanog* in ViO-ES9 cells at passage (P) 5 and 7 (B); ViO-ES9 and D3-ES cells were stained with antibodies to OCT-4, SOX-2, SSEA-1 and with alkaline phosphatase. Nuclei are stained with DAPI (blue) (C).

We then cultured a cohort (n = 15) of 2-cell embryos from the 0.3 M cohort to the blastocyst stage, to determine whether they could give rise to viable ESCs (see Fig. 1). Whole blastocyts were plated onto a monolayer of MEFs to promote their expansion and to produce colonies of cells that resembled pluripotent ESCs (Fig. 3A), which were then further passaged to demonstrate their ability to generate proliferative colonies. In all, 9 lines were established and one was fully characterised, ViO-ES9. ViO-ES9 was analysed for expression of genes associated with pluripotency. They were positive for the expression of *Oct4*, *Dppa3*, *Dppa5* and *Pramel7* but failed to express *Nanog* (Fig. 3B). Using immunocytochemistry (ICC), we also confirmed that ESC colonies contained a few isolated areas of positive staining for NANOG but no staining for OCT4, SSEA-1 or alkaline phosphatase (Fig. 3C). In contrast, D3 (control) cells exhibited positive staining for NANOG, OCT4, SSEA-1 and alkaline phosphatase in most cells (Fig. 3C).

**Figure 4:**
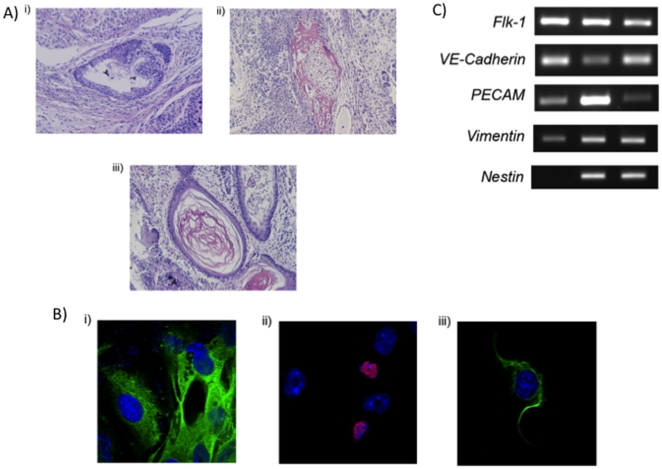
Histology and Hematoxylin and Eosin staining of teratoma tissue from ViO-ES9 cells showing differentiation into tissues indicative of the three germ layers (A) including secretory epithelium (i), articular cartilage (ii) and keratinized epithelium (iii). Immunoflorescent analysis of differentiation into all three germ layers (B): AFP (i); GATA-4 (ii); and NESTIN (iii). Secondary antibodies were labelled with Alexa Fluro® 488 (green) except for GATA-4 which was labelled with Alexa Fluro® 594 (red). Nuclei are stained with DAPI (blue). RT-PCR for Flk-1, VE-Cadherin, PECAM, Vimentin and Nestin (C).

Nevertheless, we further tested the pluripotent potential of the ViO-ES9 cells in vivo by generating teratomas. Undifferentiated cells were injected into the hind leg of SCID mice and, after 6 weeks, teratomas were recovered. Heamatoxylin and eosin staining revealed that they contained tissues indicative of the three germ layers including secretory epithelium (endoderm), cartilage (mesoderm) and epidermal tissue (ectoderm) (Fig. 4Ai–iii). The ViO-ES9 cells were also differentiated using the hanging droplet method to produce embryoid bodies, which were plated onto culture dishes to promote further differentiation. The outgrowths showed clear indications of differentiating cells and they were analysed by RT-PCR and ICC for markers of each of the 3 germ layers. Colonies were positive for endoderm (AFP – Fig. 4Bi), ectoderm (Nestin – Fig. 4Biii; Fig. 4C; Vimentin – Fig. 4C), and mesoderm (GATA4 – Fig. 4Bii; Flk-1, VE-Cadherin, PECAM – Fig. 4C).

**Figure 5:**
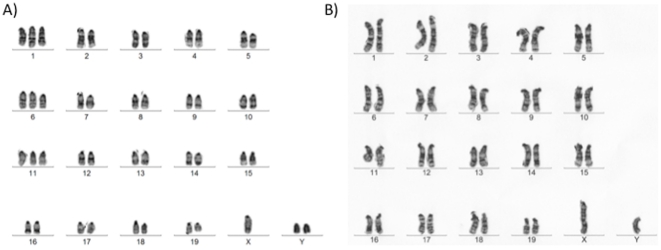
Karyotype analysis of ES cells and live offspring derived from vitrified oocytes. A) Karyotype analysis performed on ViO-ES9 cells revealed a number of abnormalities. The male cell line has a chromosome count of 46. B) Karyotype analysis performed on the mice that were generated from vitrified oocytes revealed normal karyotypes, a representative karyotype for one of the male mice shows a normal 40 XY chromosome count with no abnormalities.

Close inspection of the *in vitro* growth patterns within the culture dish of late passage cells led us to analyse the karyotype of these cells. This was abnormal (data not shown). Subsequent analysis of early passage cells revealed the same outcome (Fig. 5). Cells were commonly trisomies for chromosomes 1, 6 and 11 and contained an extra Y chromosome.

In order to determine whether the abnormal karyotype resulted from the vitrification protocol or the in vitro culture environment, we transferred embryos from fresh and the 0.1 and 0.3 M trehalose treated cohorts of oocytes to surrogates. Live offspring were generated from each cohort (see Table 2). Although higher numbers of blastocysts were obtained following IVF with fresh oocytes (Table 1), no significant differences were observed for offspring rates following embryo transfer (Table 2). The vast majority of these offspring generated from the 0.1 M and 0.3 M trehalose-treated oocytes (9/10) were karotypically normal (Fig. 5B). As one of these animals was karyotypically abnormal, we decided to further test whether vitrification was responsible for the chromosomal instability. Here, we again used fertilised fresh and vitrified oocytes from the 0.3 M cohort to produce 2-cell embryos, which were vitrified and stored for a minimum of one month (Table 3). Following warming, 2-cell embryos were transferred to surrogates who gave birth to live offspring (Table 3). None of the 3 offspring analysed showed any chromosomal instability (data not shown), although one offspring was larger than the others at birth and remained so during adolescence and adulthood. It subsequently died at 7 months whilst the others appeared healthy up to the age of 12 months at which point they were humanely killed. Furthermore, in order to determine whether the offspring derived from oocyte vitrification had normal reproduction capability, we mated control and 0 and 0.1 M cohort males with C57Bl6/J females. This resulted in the generation of live offspring at a rate of 6.14, 6.25 and 5.5 offspring per mating, respectively.

**Table 2:** Survival, and live offspring rates for vitrified oocytes.

Treatment(M of Trehalose)	VitrifiedOocytes	Post-warming survival	Two-cell(%)	Transferred	Pups (%)
Fresh	20 (not vitrified)	N/A	14 (70.0)	14	4 (20)
0	49	39 (79.6)	29 (59.2)	23 (46.9)	3 (13.0)
0.1	52	37 (71.2)	31 (59.6)	22 (42.3)	6 (27.3)
0.3	48	35 (72.9)	25 (52.1)	10 (37.5)	1 (10.0)

Fisher's test indicated no significant differences between the treatments within same column (P>0.05). Oocyte and live offspring data collected from 5 and 2 replicates respectively.

**Table 3:** Survival, vitrified two-cell embryo and live offspring rates from either fresh or vitrified oocytes.

Treatment	Vitrified embryos two-cell stage	Post-warming survival	Transferred	Pups (%)
Fresh (not vitrified)	47	22 (46.8)	16 (72.7)	6 (37.5)
0.3 M Trehalose	27	13 (48.1)	8 (61.5)	3 (37.5)

Fisher's test indicated no significant differences between the treatments within same column (P>0.05).

## Discussion

Vitrification offers the opportunity for the long-term storage of oocytes and we have demonstrated the cryoprotective potential of administering extracellular trehalose on mouse oocytes during vitrification and warming. Viability was demonstrated by oocyte post-warming survival, cleavage, pre- and post-implantation embryo development and through the generation of live offspring. We have further demonstrated that two-cell embryos from vitrified oocytes can successfully undergo a further round of vitrification and subsequent warming and give rise to live offspring. These offspring appeared to have normal reproductive function as they produced viable offspring. However, vitrified oocytes that are warmed, fertilised and then cultured to the final stages of preimplantation development had a tendency to genetic instability as demonstrated by the chromosomal aberrations observed in the ES cells derived from their blastocysts.

There is a substantial body of evidence to suggest that temperature [23] and osmotic changes [24] during the freezing and thawing process can damage the structural integrity of cells. Oocyte cryopreservation has also been reported to damage the mitotic spindle [14], harden the zona pellucida [13] and to promote oocyte activation, which leads to parthenogenesis [12]. The osmolyte, trehalose, has been used to protect mammalian cells, gametes and embryos following freezing or storage in the dry state. Addition of trehalose to a cryoprotective solution significantly improved the colony forming units of haemotopoeitic cells following thawing and culture [25]. Similarly, extracellular trehalose has been reported to either maintain or increase the post-thaw motility of boar [26], bovine [27] and ram sperm [28]. Mouse embryo viability was reported to be double when frozen in the presence of trehalose rather than in its absence [29]. Furthermore, Guo et al. showed that human primary fibroblasts, which were engineered to produce this saccharide, were viable following rehydration after storage of up to five days at the dry stage. Likewise, mouse [30] and rhesus macaque [31] sperm have been reported to promote fertilisation and embryo development following ICSI.

In our work, the post-warming survival rates were not significantly different when oocytes were vitrified in 0 (85.4%), 0.1 (82%) or 0.3 M (85.9%) trehalose but survival was significantly lower when vitrified in the 0.2 M solution (37.2%). Trehalose is understood to be a non-toxic osmolyte and the non-linear dose response of oocytes to 0.2 M solution observed in our studies was not expected. Consequently, we assessed mitochondrial clustering in each of the cohorts at this stage where we know from previous work that mitochondrial content in oocytes is a key criterion associated with developmental competency [16], [32], [33]. We report here that the addition of 0.1 M trehalose to the CPA improves the distribution of mitochondrial clustering, but 0.2 M trehalose has a harmful effect as evidenced by poor clustering and the increased presence of cytoplasmic-like vacuoles (cf Fig. 2A,B,C and D). However, increasing the trehalose concentration to 0.3 M appeared to partially restore mitochondrial clustering (Fig. 2E). These outcomes suggest an ‘all or none’ effect whereby the lower 0.1 M trehalose concentration is sufficiently low not to be harmful to mitochondria, but 0.3 M is present in sufficient abundance to have a protective effect. Consequently, the 0.2 M trehalose concentration appears to negate this protective effect.

Mitochondria are sensitive organelles with their viability dependent on a high mitochondrial membrane potential. Indeed, mitochondrial dysfunction in the oocyte is responsible for the early arrest of preimplantation embryos *in vitro* [34] and when mediated through disruption to the mitochondrial membrane potential can result in apoptosis [35], which would account for some of the embryo loss during preimplantation development. Furthermore, the failure of metaphase II oocytes to establish mitochondrial developmental competency, and then supplemented with divergent populations of mitochondria, can result in a series of disorders, such as pervasive development disorder, and genetic instability as evidenced by Turner's (XO) syndrome [36] and hence the genomic instability we observe in our ESCs.

Cleavage rates for our vitrified oocytes (50–74.4%) following IVF were not significantly different to the control IVF cohort (76.4%). Similar cleavage rates have been reported for oocytes cryopreserved in the presence of 0.5 extracellular trehalose (63%; [8]), or when slowly frozen using a non-programmable freezer in a CJ2 solution containing 18% fetal bovine serum, 1.5 M propanediol and 0.1 M sucrose, and following laser ablation of the zona pellucida prior to IVF (48%; [9]) but lower than those when trehalose was microinjected into the oocyte prior to cryopreservation (83%; [8]). Nevertheless, following warming, sperm were capable of penetrating the zona pellucida of our vitrified oocytes, fertilisation and promoting embryo development to the hatched stage without having to perform ICSI or zona laser ablation. Our developmental rates (number of blastocysts per two-cell stage embryo) were 52.4, 80.5, 29.4 and 78.0% from oocytes vitrified in the presence of 0 M, 0.1 M, 0.2 M and 0.3 M trehalose, respectively.

Following embryo transfer, there were no significant differences in offspring survival rates between non-vitrified and 0.1 M trehalose vitrified oocytes (20% v 27.3%). However, oocytes vitrified in 0 or 0.3 M trehalose resulted in 13% and 10% live offspring, respectively. These rates are similar to those reported for oocytes cryopreserved by slow cooling with intra- and extracellular trehalose [8]. Following IVF and embryo transfer, these workers reported the birth of five pups (23%) from non-cryopreserved control oocytes and 4 (19%) from cryopreserved oocytes when embryos were transferred at the two-cell stage. Similar results were reported by these workers when they transferred blastocysts from unfrozen (7 pups; 28%) and frozen oocytes (3 pups; 14%) into surrogates. In contrast, live offspring rates of 26.7% and 46.7% have been reported from oocytes cryopreserved in a choline based solution with 1.5 M 1,2-propanediol and 0.1 M sucrose, and using a programmable or non-programmable freezer, respectively and subjected to laser ablation of the zona pellucida prior to IVF [9]. Nevertheless, the live offspring generated from trehalose-vitrified oocytes maintained genetic stability as evidenced by their normal karyotypes.

Our data would clearly suggest that there is a developmental advantage for fertilised vitrified oocytes being transferred at the 2-cell rather than the blastocyst stage. The chromosomal aberrations that we observed in early and later passage ESCs were most likely the result of in vitro culture to the blastocyst stage followed by ESC derivation. This also seemed to affect their ability to maintain pluripotent gene expression although they produced teratomas and representative gene expression of markers of differentiation. It is thus highly likely that the uterus of the surrogate mouse offers considerable metabolic support that the in vitro culture environment is unable to provide. Indeed, in vitro culture can result in a variety of genetic and epigenetic abnormalities [37]. Similar outcomes arise from poor diet prior to conception and during pregnancy, which affect epigenetic integrity, offspring size and development rates [38].

Nevertheless, it is likely that the inter-uterine environment of healthy and reproductive competent mice will be better able to sustain and support the metabolic function of these embryos during early development and until the embryonic genome takes over [39] and mitochondrial replication is initiated at the blastocyst stage [16]. Consequently, key molecular events such as cytokinesis, karyokinesis and *de novo* methylation can be appropriately metabolically supported. The current in vitro culture systems are likely to be unable to provide this degree of metabolic support. This is further substantiated by the generation of live offspring from the vitrification of 2-cell embryos generated from vitrified oocytes, that were able to, on the whole, maintain genetic integrity and give rise to live offspring. This, to the best of our knowledge, is the first report of such an outcome and should eliminate any concerns that vitrification may raise for subsequent offspring survival. However, endeavours to generate pluripotent embryonic stem cells from vitrified oocytes to provide multiple genetically identical donors for nuclear transfer will require refined culture systems to ensure that genetic abnormalities can be avoided and that these cells offer a viable approach to rescue endangered species.

A further concern regarding vitrification has been the potential for cross-contamination. Vitrification of oocytes and embryos is traditionally carried out in open systems such as open pulled straws [40], electron microscope grids [41], cryoloop [42], solid-surface vitrification [43] or nylon mesh [44]. The use of these systems subjects the oocytes or embryos to direct contact with liquid nitrogen and to the risk of contamination as it is widely acknowledged that viral and bacterial pathogenic agents survive in liquid nitrogen [45]. Bielanski et al [46] observed that, when samples are stored in sealed straws or closed cryovials, the risk of pathogen contamination during vitrification and storage of samples in liquid nitrogen is removed. They reported that, when samples were stored in unsealed containers, the incidence of viral contamination due to exposure to contaminated liquid nitrogen was 22% whilst no contamination was observed in samples stored in closed containers. Therefore, the method presented here reduces this risk, as straws are sealed with a metal sealing ball before vitrification is carried out. Indeed, use of the method presented here together with the use of sterilised liquid nitrogen either by UV exposure [47] and storage in vapour phase tanks [48] will assist in avoiding the risk of pathogen cross-contamination during sample storage.

In conclusion, we have shown that oocytes can be successfully vitrified using a combination of trehalose and sucrose based CPAs and that successful vitrification using trehalose is concentration-dependent. The use of 0.1 and 0.3 M trehalose provides the oocyte with mitochondrial protection and ensures that, when embryos are transferred at early stages of development, live offspring can be successfully produced. Furthermore, this form of oocyte storage can be used as part of a ‘closed’ vitrification system and will prevent cross-contamination.

## Materials and Methods

Experimental procedures were approved by the Monash Medical Centre Animal Ethics Committee for animal experimentation and were conducted in accordance with the Australian National Health and Medical Research Council (NHMRC) guidelines. Approval numbers were MMCA 2007/1 and MMCA 2007/39.

### Mice

F1 hybrid mice (C57Bl×CBA) were used for the present study. Mice were kept under constant environmental conditions of 12L∶12D and a temperature of 25°C.

### Media

All media were prepared from analytical grade chemicals (BDH Chemicals Pty., Ltd., Melbourne, Australia or Sigma-Aldrich Co., St. Louis, MO). Oocytes were collected in modified KSOM-HEPES and cultured in modified KSOMaa [49]. Embryos were also cultured in modified KSOMaa. Sperm were collected and capacitated in modified Tyrode's medium (mT6; [50], [51]) without sodium pyruvate or sodium lactate.

### Oocyte collection

Six-week-old females were superovulated by an intraperitoneal injection of 5 IU equine chorionic gona-dotrophin (Folligon, Intervet International, Boxmeer, The Netherlands) followed by 5 IU hCG (Chorulon, Intervet International) in 0.2 ml saline 48 h later. At 13 to 14 h after hCG injection, oocyte-cumulus complexes were collected from the oviducts into KSOM-HEPES containing 60 IU/ml hyaluronidase (Sigma type IV-S) for 5 min. Adhering cumulus cells were removed from oocytes by gentle pipetting and washed in KSOM-HEPES. Oocytes were then placed into 20 µl drops of modified KSOMaa previously equilibrated under mineral oil in 5% CO_2_ in air at 37°C for at least 30 min before they were vitrified or placed into a 90 µl drop of mT6 under oil in 5% CO_2_ in air for 30 min before IVF was carried out.

### Oocyte/Embryo Vitrification and Warming

Exposure of oocytes and embryos to cryoprotectants was carried out on the microscope stage set to 37°C. Cohorts of oocytes or two-cell embryos (5–10), as indicated, were placed into 20 µL drops of KSOM-Hepes containing 8% DMSO (1.4 M) and 8% (1.4 M) ethylene glycol for 1 min at 37°C. Oocytes and two-cell embryos were then transferred to KSOM-HEPES containing 16% DMSO (2.8 M) and 16% ethylene glycol (2.8 M), 10 mg/ml Ficoll, 0.65 M sucrose with 0, 0.1, 0.2 or 0.3 M trehalose for 20 s at 37°C. Oocytes were then loaded into 0.25 ml straws sealed with a metal sealing ball (Minitube, Smythesdale, VIC, Australia) slowly pressed against a metal block (Cryologic Vitrification block CVM); Cryologic, Mulgrave, VIC, Australia) for 30–45 s and then transferred into liquid nitrogen and stored for at least a week in a liquid nitrogen vapour phase tank.

### Warming

Straws were removed from the storage tank and transferred into a container with liquid nitrogen. Before warming, straws were held for 30–40 sec in the vapour phase of liquid nitrogen, held in the air for 5 sec then transferred into a water bath at 37°C. The straws were then dried, the sealing ball removed and the oocytes expelled into a 20 µl drop of KSOM-Hepes containing 0.25 M sucrose at 37°C for 2 min. The oocytes were then transferred to a 20 µl drop of KSOM-Hepes containing 0.125 M sucrose at 37°C for a further 3 min, then into KSOM-Hepes at 37°C for 5 min and finally into a 90 µl drop of mT6 under oil in 5% CO_2_ in air for 30 min before IVF was carried out.

### Oocyte imaging

MitoTracker Deep Red FM (Invitrogen) was used to visualize oocyte mitochondria. Zona pellucidae were first removed by incubation in an acidified Tyrode's solution for 3–5 minutes [52]. After recovery, zona-free oocytes were incubated in KSOM-Hepes containing 100 nM of Mitotracker Deep Red for 45 min. Oocytes were then washed twice with PBS containing 0.05% (v/v) Tween-20 (PBS-T) and fixed in 2% (w/v) paraformaldehyde in PBS-T. Following fixation, zona-free oocytes were transferred to an 8-well slide (Ibidi) for analysis with a Nikon C1 laser scanning confocal microscope on a Nikon Ti-E inverted microscope base. 60×1.4NA objective was used to image samples. 633 nm laser was used to specifically excite MitoTracker Deep Red and the fluorescence emission was detected via a 645/75 nm filter.

### Ripley's clustering analysis

Ripley's K-function, for three-dimensional data, weighted by the intensity of point:
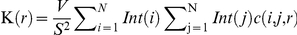
The intensity of a data point x (given by Int(x)) was considered to be the assumed (relative) number of actual agents (or points) in that position. V is the volume of the domain, N is the number of data points, and S is the sum of all intensities: 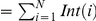, i.e: the total number of assumed agents. c(i,j,r) is an indicator function, which obtains the value = 1 if the distance between the i'th and j'th points is less or equal to r, and = 0 otherwise.

For a data set in a three dimensional space, the benchmark obtained by a homogeneous Poisson process is: 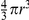. Values significantly different to it will indicate the points are not distributed homogenously, i.e. the points are spatially clustered.

In order to avoid underestimation of K due to edge effects we introduced a buffer zone, so that the first sum would only go over points for which the distance from any of the domain's boundaries was larger or equal to the largest value of r.

### In vitro fertilization (IVF)

Sperm were collected from 10 to 12-week-old males. Briefly, both caudae epididymides were dissected from the mice and a small slit was made in each cauda before transfer to 1 ml modified mT6 and equilibrated in 5% CO_2_ in air at 37°C in a 5 ml (NUNC, Roskilde, Denmark) under oil. The epididymal tissue was then removed from the tube 1 h later, and the sperm concentration was assessed and adjusted to 3 to 5×10^6^ sperm/ml and a 10 µl aliquot was added to the oocytes. Sperm and oocytes were incubated for 6 h, under mineral oil. Oocytes were then removed from the sperm solution, washed in KSOM-Hepes, and cultured at 37°C in modified KSOMaa under mineral oil in 5% CO_2_ in air. Six hours later, oocytes were examined for signs of normal fertilization. Oocytes with two pronuclei and a second polar body were regarded as fertilized. These oocytes were separated from unfertilized oocytes and cultured in modified KSOMaa at 37°C under 5% CO in air up to 96 h for development to the blastocyst stage.

### Parthenogenic activation

Twenty to twenty five freshly collected MII oocytes and vitrified oocytes per cohort were thawed and transferred into a 90 µl drop of modified Tyrode's medium under oil in 5% CO_2_ in air for 6 h without sperm and cultured in modified KSOMaa at 37°C under 5% CO_2_ in air for up to 96 h.

### Embryo transfers

Two-cell embryos produced following IVF were transferred to the oviducts of Day 1 pseudopregnant F1 females mated to vasectomized F1 males of proven sterility. Between 8 and 12 embryos were transferred to each oviduct. Females were allowed to give birth and the number of pups on the day of birth was recorded.

### Natural mating

Two males from the breeding colony (control) and from the 0 and 0.1 cohorts, were bred for two cycles with C57Bl6/J six week old females and the number of live offspring per male/treatment recorded.

### Culture and preparation of feeder layers

Murine embryonic fibroblasts (MEFS) were cultured in High Glucose Dulbecco's Modified Eagle's Medium (DMEM; Sigma, Gillingham, UK) with 10% fetal bovine serum (FBS; Sigma), 2 mM L-glutamine (Invitrogen Life Technologies, Paisley, UK), 1% non-essential amino acids (NEAA; Invitrogen Life Technologies) and 1% penicillin/streptomycin solution (Sigma). MEFs were inactivated using mitomycin C (Sigma; 10 µg/ml) for 2 hours at 37°C/5% CO_2_, and then washed 3 times with PBS (Sigma) and cultured overnight or frozen before use as feeder cells for undifferentiated mES cells.

### Derivation of mES cells

Expanded blastocysts were cultured until they hatched. They were then transferred to 96-well plates containing MEFS and blastocyst culture media and allowed to expand. Once an expanding colony had been identified, the cells were cultured as for D3 mES cells. They were manually passaged onto 48-, 24- and 6-well plates (BD Falcon, North Ryde, NSW, Australia) containing MEFS, and once established, onto Petri-dishes (BD Falcon).

### Culture of mES cells

D3 mES cells and ViO- ViO-ES9s were cultured, as described in [53]. Briefly they were placed in High Glucose DMEM (Sigma) with 15% ES cell screened fetal bovine serum (FBS; Hyclone, Utah, US), 1% penicillin/streptomycin solution (Invitrogen Life Technologies), 2 mM L-glutamine (Invitrogen Life Technologies), 1% NEAA (Invitrogen Life Technologies), 0.1 mM β-mercaptoethanol (Sigma) and 1000 U/ml LIF.

### Differentiation of mESCs

Undifferentiated ViO-ES9 and D3 mES cells were induced to differentiate using the hanging drop method [54]. Briefly, mES cells (Day 0) were dissociated with 0.25% trypsin-EDTA (Sigma) for 2 minutes, resuspended into mES cell media and plated as 20 µl droplets (approximately 450 cells per drop) on the lid of an inverted Petri dish (Sterilin, Staffordshire, UK) for 48 hours (Days 1 to 2) to promote the formation of EBs. The EBs were then placed into suspension for a further 5 (Days 3 to 7, D3 mES cells) or 6 days (Days 3 to 8, R1 mESCs) at 37°C/5%CO_2_. The EBs were then plated onto gelatin (0.1%; Sigma) coated 6 well plates (Nunc, Roskilde, Denmark) and cultured up to Day 20 of differentiation.

### RNA isolation

RNA was extracted from cells and contaminating DNA eliminated using the RNAqueous®-4PCR Kit (Applera, Warrington, U.K., http://www.appliedbiosystems.com) according to the manufacturer's protocol for mammalian cells.

### Reverse Transcription

cDNA was produced using the Bioscript™ system (Bioline, London, U.K., http://www.bioline.com) according to the manufacturer's protocol. Each reaction contained 5 µg RNA and 200 U of reverse transcriptase. The reaction was performed at 42°C for 1 h in a MJ Research PTC-200 thermocycler (GRI, Braintree, UK).

### PCR analysis

PCR was performed in 50 µl reactions containing 200 ng of cDNA, 1× PCR buffer (Bioline, London, UK), 1.5 mM MgCl_2_ (Bioline), 100 µM dNTPs (Bioline), 0.5 µM for each of the forward and reverse primers (Gapdh: GGGAAGCCCATCACCATCTTC and AGAGGGGCCATCCACAGTCT to produce 366 bp; Oct4: AGTATGAGGCTACAGGGACA and CAAAGCTCCAGGTTCTCTTG - 260 bp; Dppa3: CTTTCCCAAGAGAAGGGTCC and TGCAGAGACATCTGAATGGC - 149 bp; Dppa5: GCTTGATCTCGTCTTCCCTG and TCCATTTAGCCCGAATCTTG - 293 bp; Pramel7: AGAGAACCCACATGGCTTTG and GGATTTGGCTTGGCATACAT - 336 bp; Nanog: AGGGTCTGCTACTGAGATGCTCTG and CAACCACTGGTTTTTCTGCCACCG - 364 bp; Flk-1: TCTGTGGTTCTGCGTGGAGA and GTATCATTTCCAACCACCCT - 270 bp; *CD31*: 5′-CGCACCTTGATCTTCCTTTC and AAGGCGAGGAGGGTTAGGTA - 244 bp; and *VE-cadherin*: TCCTCTGCATCCTCACCATCACA and GTAAGTGACCAACTGCTCGTGAAT - 122 bp and 2.5 U *BioTaq* DNA polymerase (Bioline). Reactions were run on a MJ Research PTC-200 machine: initial denaturation at 95°C for 5 min; followed by 35 cycles of 94°C for 30 sec (except for Gapdh 25 cycles); annealing at 60°C for 45 sec (Gapdh; Flk-1), 55°C for 30 sec (Oct4), 57°C for 30 secs (VE-Cadherin), 60°C for 30 sec (Dppa3), 60°C for 30 sec (Dppa5), 60°C for 45 sec (Pramel7), and 62°C for 20 sec (Nanog); 72°C for 45 sec; and a final extension at 72°C for 3 min. PCR products were resolved on 2% agarose gels (Bioline) at 100 V for 1 h.

### Karyotype analysis

Karyotype analyses of both mice and ES cells were performed by Southern Cross Pathology, Australia. In brief, for mice primary tail tip fibroblasts were isolated following mechanical dissection and incubated in trypsin for 7 minutes and cultured in MEF media until cell explants were observed. ES cells in their exponential growth phase were analysed.

### Teratoma generation

To examine teratoma formation, 1–2×10^6^ cells were injected into the rear leg muscle of 4–6 week old severe combined immunodeficient (SCID) mice. After 4 weeks, the teratomas were excised and fixed in 4% paraformaldehyde, embeded in paraffin, sectioned at 5 uM and stained with Hematoxylin and Eosin using standard methods performed by the Monash Institute of Medical Research Histology Core Facility.

### Statistical analyses

Survival, embryo development and offspring data were subjected to contingency tables and Fisher's exact test. For oocyte volume and mitochondrial clustering measurements, the means of each group were tested against the control (Fresh oocytes) by One-Way ANOVA test with Dunnett's Multiple Comparison post-test.

## References

[1] Tao T, Del Valle A (2008). Human oocyte and ovarian tissue cryopreservation and its application.. J Assist Reprod Genet.

[2] Jain JK, Paulson RJ (2006). Oocyte cryopreservation.. Fertil Steril.

[3] Ambrosini G, Andrisani A, Porcu E, Rebellato E, Revelli A (2006). Oocytes cryopreservation: state of art.. Reprod Toxicol.

[4] Bowles EJ, Campbell KH, St John JC (2007). Nuclear transfer: preservation of a nuclear genome at the expense of its associated mtDNA genome(s).. Curr Top Dev Biol.

[5] Evans MJ, Kaufman MH (1981). Establishment in culture of pluripotential cells from mouse embryos.. Nature.

[6] Campbell KH, McWhir J, Ritchie WA, Wilmut I (1996). Sheep cloned by nuclear transfer from a cultured cell line.. Nature.

[7] Wilmut I, Schnieke AE, McWhir J, Kind AJ, Campbell KH (1997). Viable offspring derived from fetal and adult mammalian cells.. Nature.

[8] Eroglu A, Bailey SE, Toner M, Toth TL (2009). Successful cryopreservation of mouse oocytes by using low concentrations of trehalose and dimethylsulfoxide.. Biol Reprod.

[9] Stachecki JJ, Cohen J, Schimmel T, Willadsen SM (2002). Fetal development of mouse oocytes and zygotes cryopreserved in a nonconventional freezing medium.. Cryobiology.

[10] Kazem R, Thompson LA, Srikantharajah A, Laing MA, Hamilton MP (1995). Cryopreservation of human oocytes and fertilization by two techniques: in-vitro fertilization and intracytoplasmic sperm injection.. Hum Reprod.

[11] Mavrides A, Morroll D (2005). Bypassing the effect of zona pellucida changes on embryo formation following cryopreservation of bovine oocytes.. Eur J Obstet Gynecol Reprod Biol.

[12] Van der Elst J, Van den Abbeel E, Nerinckx S, Van Steirteghem A (1992). Parthenogenetic activation pattern and microtubular organization of the mouse oocyte after exposure to 1,2-propanediol.. Cryobiology.

[13] Vincent C, Pickering SJ, Johnson MH (1990). The hardening effect of dimethylsulphoxide on the mouse zona pellucida requires the presence of an oocyte and is associated with a reduction in the number of cortical granules present.. J Reprod Fertil.

[14] Sathananthan AH, Ng SC, Trounson AO, Bongso A, Ratnam SS (1988). The effects of ultrarapid freezing on meiotic and mitotic spindles of mouse oocytes and embryos.. Gamete Res.

[15] Bouquet M, Selva J, Auroux M (1993). Cryopreservation of mouse oocytes: mutagenic effects in the embryo?. Biol Reprod.

[16] Spikings EC, Alderson J, St John JC (2007). Regulated mitochondrial DNA replication during oocyte maturation is essential for successful porcine embryonic development.. Biol Reprod.

[17] Wilding M, Dale B, Marino M, di Matteo L, Alviggi C (2001). Mitochondrial aggregation patterns and activity in human oocytes and preimplantation embryos.. Hum Reprod.

[18] Cohen J, Scott R, Schimmel T, Levron J, Willadsen S (1997). Birth of infant after transfer of anucleate donor oocyte cytoplasm into recipient eggs.. Lancet.

[19] Gardner DK, Lane M (1997). Culture and selection of viable blastocysts: a feasible proposition for human IVF?. Hum Reprod Update.

[20] Lane M, Gardner DK (2001). Vitrification of mouse oocytes using a nylon loop.. Mol Reprod Dev.

[21] Crowe LM, Mouradian R, Crowe JH, Jackson SA, Womersley C (1984). Effects of carbohydrates on membrane stability at low water activities.. Biochim Biophys Acta.

[22] Kasai M, Niwa K, Iritani A (1980). Survival of mouse embryos frozen and thawed rapidly.. J Reprod Fertil.

[23] Coticchio G, Bonu MA, Borini A, Flamigni C (2004). Oocyte cryopreservation: a biological perspective.. Eur J Obstet Gynecol Reprod Biol.

[24] Mullen SF, Agca Y, Broermann DC, Jenkins CL, Johnson CA (2004). The effect of osmotic stress on the metaphase II spindle of human oocytes, and the relevance to cryopreservation.. Hum Reprod.

[25] Zhang XB, Li K, Yau KH, Tsang KS, Fok TF (2003). Trehalose ameliorates the cryopreservation of cord blood in a preclinical system and increases the recovery of CFUs, long-term culture-initiating cells, and nonobese diabetic-SCID repopulating cells.. Transfusion.

[26] Gutierrez-Perez O, Juarez-Mosqueda Mde L, Carvajal SU, Ortega ME (2009). Boar spermatozoa cryopreservation in low glycerol/trehalose enriched freezing media improves cellular integrity.. Cryobiology.

[27] Foote RH, Chen Y, Brockett CC, Kaproth MT (1993). Fertility of bull spermatozoa frozen in whole milk extender with trehalose, taurine, or blood serum.. J Dairy Sci.

[28] Sanchez-Partida LG, Setchell BP, Maxwell WM (1998). Effect of compatible solutes and diluent composition on the post-thaw motility of ram sperm.. Reprod Fertil Dev.

[29] Honadel TE, Killian GJ (1988). Cryopreservation of murine embryos with trehalose and glycerol.. Cryobiology.

[30] McGinnis LK, Zhu L, Lawitts JA, Bhowmick S, Toner M (2005). Mouse sperm desiccated and stored in trehalose medium without freezing.. Biol Reprod.

[31] Sanchez-Partida LG, Simerly CR, Ramalho-Santos J (2008). Freeze-dried primate sperm retains early reproductive potential after intracytoplasmic sperm injection.. Fertil Steril.

[32] Santos TA, El Shourbagy S, St John JC (2006). Mitochondrial content reflects oocyte variability and fertilization outcome.. Fertil Steril.

[33] El Shourbagy SH, Spikings EC, Freitas M, St John JC (2006). Mitochondria directly influence fertilisation outcome in the pig.. Reproduction.

[34] Thouas GA, Trounson AO, Jones GM (2006). Developmental effects of sublethal mitochondrial injury in mouse oocytes.. Biol Reprod.

[35] Ramalho-Santos J, Varum S, Amaral S, Mota PC, Sousa AP (2009). Mitochondrial functionality in reproduction: from gonads and gametes to embryos and embryonic stem cells.. Hum Reprod Update.

[36] Barritt JA, Brenner CA, Malter HE, Cohen J (2001). Rebuttal: interooplasmic transfers in humans.. Reprod Biomed Online.

[37] Doherty AS, Mann MR, Tremblay KD, Bartolomei MS, Schultz RM (2000). Differential effects of culture on imprinted H19 expression in the preimplantation mouse embryo.. Biol Reprod.

[38] Ashworth CJ, Toma LM, Hunter MG (2009). Nutritional effects on oocyte and embryo development in mammals: implications for reproductive efficiency and environmental sustainability.. Philos Trans R Soc Lond B Biol Sci.

[39] Bolton VN, Oades PJ, Johnson MH (1984). The relationship between cleavage, DNA replication, and gene expression in the mouse 2-cell embryo.. J Embryol Exp Morphol.

[40] Vajta G, Holm P, Kuwayama M, Booth PJ, Jacobsen H (1998). Open pulled straw (OPS) vitrification: A new way to reduce cryoinjuries of bovine ova and embryos.. Molecular Reproduction and Development.

[41] Martino A, Songsasen N, Leibo SP (1996). Development into blastocysts of bovine oocytes cryopreserved by ultra-rapid cooling.. Biology of Reproduction.

[42] Lane M, Gardner DK (2001). Vitrification of mouse oocytes using a nylon loop.. Molecular Reproduction and Development.

[43] Dinnyès A, Carolan C, Loneragan P, Solti L, Massip A (1995). In vitro survival of in vitro produced (IVP) bovine embryos frozen or vitrified by techniques suitable for direct transfer.. Theriogenology.

[44] Abe Y, Hara K, Matsumoto H, Kobayashi J, Sasada H (2005). Feasibility of a Nylon-Mesh Holder for Vitrification of Bovine Germinal Vesicle Oocytes in Subsequent Production of Viable Blastocysts.. Biology of Reproduction.

[45] Fountain D, Ralston M, Higgins N, Gorlin JB, Uhl L (1997). Liquid nitrogen freezers: a potential source of microbial contamination of hematopoietic stem cell components.. Transfusion.

[46] Bielanski A, Nadin-Davis S, Sapp T, Lutze-Wallace C (2000). Viral contamination of embryos cryopreserved in liquid nitrogen.. Cryobiology.

[47] Parmegiani L, Accorsi A, Cognigni GE, Bernardi S, Troilo E (2010). Sterilization of liquid nitrogen with ultraviolet irradiation for safe vitrification of human oocytes or embryos.. Fertil Steril.

[48] Cobo A, Romero JL, Perez S, de los Santos MJ, Meseguer M (2010). Storage of human oocytes in the vapor phase of nitrogen.. Fertil Steril.

[49] Biggers JD, McGinnis LK, Raffin M (2000). Amino acids and preimplantation development of the mouse in protein-free potassium simplex optimized medium.. Biol Reprod.

[50] Lacham-Kaplan O, Shaw J, Sanchez-Partida LG, Trounson A (2003). Oocyte activation after intracytoplasmic injection with sperm frozen without cryoprotectants results in live offspring from inbred and hybrid mouse strains.. Biol Reprod.

[51] Fraser LR (1984). Mouse sperm capacitation in vitro involves loss of a surface-associated inhibitory component.. J Reprod Fertil.

[52] Nicolson GL, Yanagimachi R, Yanagimachi H (1975). Ultrastructural localization of lectin-binding sites on the zonae pellucidae and plasma membranes of mammalian eggs.. J Cell Biol.

[53] Facucho-Oliveira JM, Alderson J, Spikings EC, Egginton S, St John JC (2007). Mitochondrial DNA replication during differentiation of murine embryonic stem cells.. J Cell Sci.

[54] Keller GM (1995). In vitro differentiation of embryonic stem cells.. Curr Opin Cell Biol.

